# Outcome of patients with different stages of acute-on-chronic liver failure treated with artificial liver support system

**DOI:** 10.3389/fmed.2024.1381386

**Published:** 2024-05-17

**Authors:** Yuanji Ma, Yan Xu, Lingyao Du, Lang Bai, Hong Tang

**Affiliations:** Center of Infectious Diseases, West China Hospital of Sichuan University, Chengdu, China

**Keywords:** acute-on-chronic liver failure, artificial liver support system, international normalized ratio of prothrombin time, timing, prognosis

## Abstract

**Background:**

Elevated international normalized ratio of prothrombin time (PT-INR) is one of the key characteristics of acute-on-chronic liver failure (ACLF). Whether the staging of PT-INR has the ability to screen out subgroups of ACLF patients who would be more eligible for artificial liver support system (ALSS) treatment has not been studied in detail.

**Methods:**

A previous study enrolled patients receiving ALSS treatment with regional citrate anticoagulation from January 2018 to December 2019. Patients with different PT-INR intervals were retrospectively enrolled: 1.3 ≤ PT-INR < 1.5 (Pre-stage), 1.5 ≤ PT-INR < 2.0 (Early-stage), 2.0 ≤ PT-INR < 2.5 (Mid-stage), and PT-INR ≥ 2.5 (End-stage). The Cox proportional hazards models were used to estimate the association between stages of ACLF or sessions of ALSS treatment and 90 day mortality.

**Results:**

A total of 301 ACLF patients were enrolled. The 90 day mortality risk of Early-stage ACLF patients (adjusted hazard ratio (aHR) (95% confidence interval (CI)), 3.20 (1.15–8.89), *p* = 0.026), Mid-stage ACLF patients (3.68 (1.34–10.12), *p* = 0.011), and End-stage ACLF patients (12.74 (4.52–35.91), *p* < 0.001) were higher than that of Pre-stage ACLF patients, respectively. The 90 day mortality risk of Mid-stage ACLF patients was similar to that of Early-stage ACLF patients (1.15 (0.69–1.94), *p* = 0.591). The sessions of ALSS treatment was an independent protective factor (aHR (95% CI), 0.81 (0.73–0.90), *p* < 0.001). The 90 day mortality risk in ACLF patients received 3–5 sessions of ALSS treatment was lower than that of patients received 1–2 sessions (aHR (95% CI), 0.34 (0.20–0.60), *p* < 0.001), whereas the risk in patients received ≥6 sessions of ALSS treatment was similar to that of patients received 3–5 sessions (0.69 (0.43–1.11), *p* = 0.128).

**Conclusion:**

ACLF patients in Pre-, Early-, and Mid-stages might be more eligible for ALSS treatment. Application of 3–5 sessions of ALSS treatment might be reasonable.

## Introduction

Acute-on-chronic liver failure (ACLF) is a common clinical syndrome of severe liver disease, characterized by rapid disease progression and high mortality (1, 2). The main organs or systems involved in ACLF include liver, coagulation, brain, kidney, circulation and respiration (1). Several academic organizations have put forward their own ACLF definitions, and consider that abnormal coagulation function, especially the elevated international normalized ratio (INR) of prothrombin time (PT) (PT-INR), is one of the key characteristics of ACLF (1–3). The PT-INR is closely related to disease severity and prognosis of ACLF patients (1–3), and it is one of the most important criteria for stage division of ACLF in Eastern countries, such as China (4).

At present, liver transplantation is the most effective treatment for ACLF patients (5). However, it is limited by organ scarcity and patient selection. Over the past decades, artificial liver support system (ALSS) treatment has been employed as an available treatment method for ACLF. Some studies found that ACLF patients could recover from ALSS along with standard medicine treatment (6, 7), especially those received plasma exchange (PE)-centered ALSS treatment (8–10). In recent years, PE-centered ALSS treatment has been recommended as a first line treatment method for patients with liver failure in China and USA (11, 12). Although a few studies argued that ALSS treatment might not improve prognosis of ACLF, it is still a safe, well tolerated and widely used treatment and could act as a bridge to liver transplantation until an appropriate donor liver is available (13).

One possible underlying reason for these inconsistent results is the difference in patient selection. Some studies have reported that milder ACLF patients would encounter a significantly better prognosis than those with severer disease after ALSS treatment (14, 15). Currently, several predictive models have been developed to screen out subgroups of ACLF patients who may benefit from ALSS treatment (16, 17). But these models are complicated in calculation. The PT-INR is a simple and easy-to-use indicator which is strongly related to the severity of ACLF. Whether it also has the similar ability to screen out subgroups has not been studied in detail. Hence, we conducted a secondary data analysis based on a previous study enrolled patients receiving ALSS treatment with regional citrate anticoagulation to evaluate the ability of PT-INR in identifying subgroups of ACLF patients eligible for ALSS treatment. We presented the following article in accordance with the STROBE reporting checklist.

## Methods

### Study design and patients

A secondary data analysis based on a previous study enrolled patients receiving ALSS treatment with regional citrate anticoagulation at the Center of Infectious Diseases, West China Hospital of Sichuan University between January 2018 and December 2019 was conducted to evaluate the ability of PT-INR to identify subgroups of patients with hepatitis B virus (HBV)-associated ACLF (HBV-ACLF) who would benefit from ALSS treatment. The previous study was approved by the Ethics Committee of West China Hospital of Sichuan University (No. 2020 (650)), and was registered with ChiCTR2000035013. The previous study was conducted in accordance with the Declaration of Helsinki (as revised in 2013). Written informed consent was obtained from each participant or his/her parent or legal guardian.

**Figure 1:**
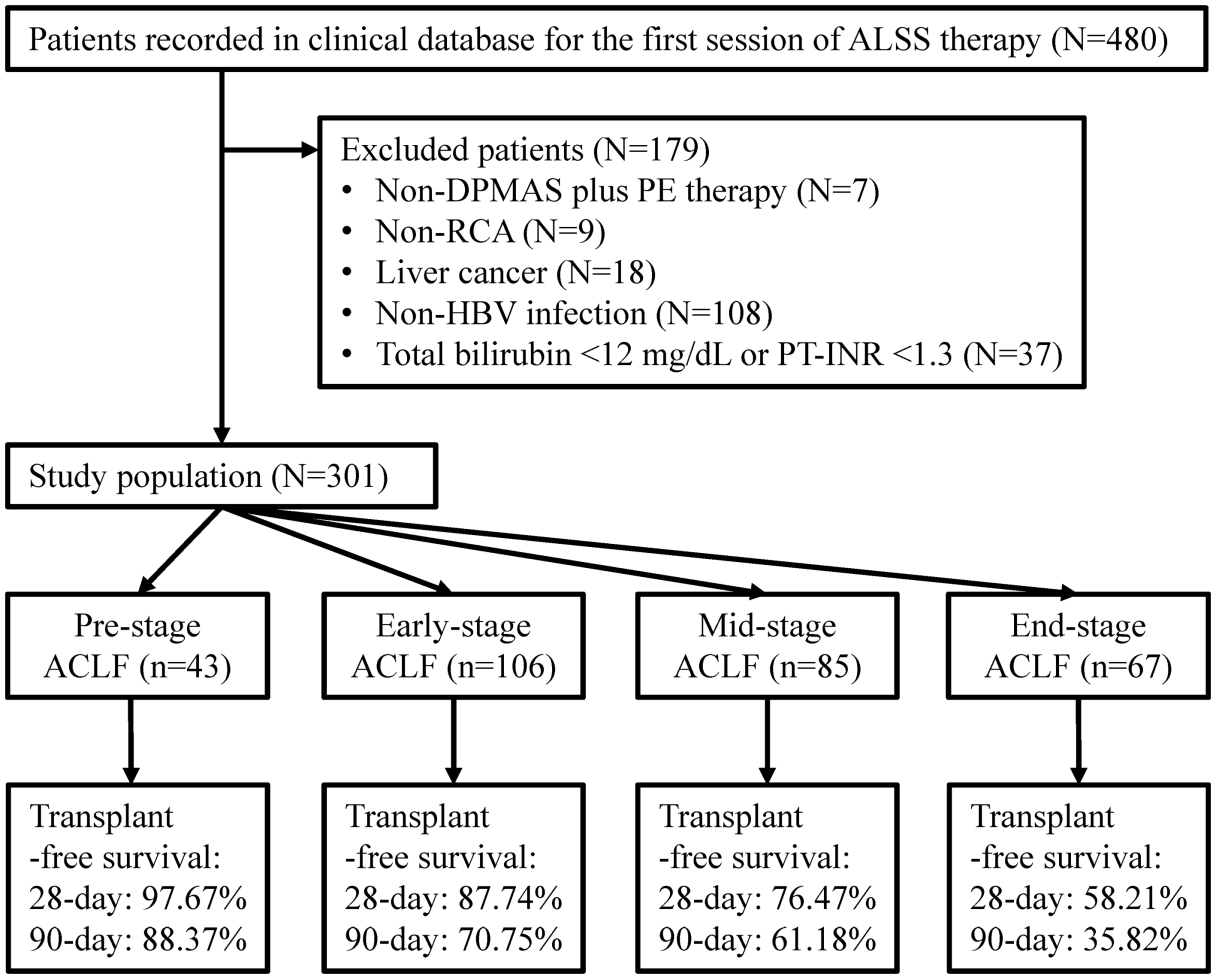
Flow diagram of patient selection. ALSS, artificial liver support system; DPMAS, double plasma molecular absorption system; PE, plasma exchange; RCA, regional citrate anticoagulation; HBV, hepatitis B virus; PT-INR, international normalized ratio (INR) of prothrombin time (PT); ACLF, acute-on-chronic liver failure.

Patients enrolled in the previous study were retrospectively screened in this study (Figure 1). Patients who had chronic HBV infection, total bilirubin ≥12 mg/dL (205 μmol/L) and PT-INR ≥ 1.5 were diagnosed with HBV-ACLF and included in the study (3). Patients with chronic HBV infection, total bilirubin ≥12 mg/dL and 1.3 ≤ PT-INR < 1.5 were diagnosed with Pre-stage HBV-ACLF and included too (18). Patients treated with non-double plasma molecular adsorption system (DPMAS) plus PE treatment or non-regional citrate anticoagulation, and patients with liver cancer or without HBV infection were excluded. The remaining patients were enrolled.

### Staging and assessment of disease

Enrolled patients were divided into 4 groups according to the different PT-INR intervals: 1.3 ≤ PT-INR < 1.5 (Pre-stage), 1.5 ≤ PT-INR < 2.0 (Early-stage), 2.0 ≤ PT-INR < 2.5 (Mid-stage), and PT-INR ≥ 2.5 (End-stage) (1, 3, 18).

The disease severity was rated according to scoring systems including Chinese Group on the Study of Severe Hepatitis B (COSSH) ACLF score, European Association for the Study of the Liver—Chronic Liver Failure (CLIF)-Consortium (CLIF-C) ACLF score, Asian Pacific Association for the Study of the Liver (APASL)—ACLF Research Consortium (AARC) score, etc. (1–3). The function of brain, kidney, circulation and respiration was assessed by CLIF-organ failure (CLIF-OF) scores (1).

### Treatment

All patients received ALSS treatment along with standard medicinal treatment. The standard medicinal treatment included management of precipitating factors, underlying chronic liver diseases, and complications. Hepatoprotective treatment and supportive treatment were also administered.

The basic techniques of non-biological ALSS treatment include PE, plasma adsorption and continuous renal replacement therapy (CRRT). PE-centered ALSS treatment is widely used in China, and the recommended dose for PE is at least 1 total plasma volume (11, 12). Due to the shortage of plasma in recent years and some studies have provided evidence that plasma adsorption plus PE treatment could reduce the amount of plasma without impairing therapeutic effect (19–22), plasma adsorption plus PE treatment with half total plasma volume (approximately 1,500 mL) is now widely used. If the patient has grade III/IV hepatic encephalopathy or has indications for CRRT treatment, CRRT is added (23, 24). In this study, all patients received plasma adsorption (namely DPMAS treatment) for 2 h followed immediately by PE treatment with 1,500 mL plasma for nearly an hour, which were the same as previously described (25). The parameters of the CRRT machine were set to a blood flow of 130 mL/min, a plasma separation flow of 1,500 mL/h, and a plasma return flow of 1,500 mL/h. The ALSS treatment was performed every 1–2 days.

ALSS treatment was discontinued due to one of the following conditions: (1) Patients refused to continue ALSS treatment, or the ones whose condition did not allow further ALSS treatment (2). Response to ALSS treatment: patient’s condition improved with total bilirubin <10 mg/dL (171 μmol/L) and decreased PT-INR (17). ALSS treatment was continued intermittently for critically ill ACLF patients who intended to receive liver transplantation in order to stabilize their condition.

### Outcome

All patients were followed up for 90 days unless death or liver transplantation. Cases were classified as dead if they received a liver transplant during follow-up. The primary outcome was 90 day mortality.

### Statistical analysis

Quantitative data are represented as median (*P*_25_–*P*_75_) and compared by Mood’s median test. Qualitative data are represented as frequencies (proportion) and compared by chi-squared test. Bonferroni method was applied for multiple comparisons. The Cox proportional hazards models were used to estimate the association between stages of ACLF or sessions of ALSS treatment and 90 day mortality. Statistical significance was set at *p* < 0.05. The statistical tests were performed using SPSS v.22 (IBM SPSS). The survival curves were drawn using SPSS v.22 (IBM SPSS), and the forest plots were drawn using GraphPad Prism 9 (GraphPad Software Inc.), respectively.

## Results

### Patient characteristics

A total of 301 patients were enrolled (Figure 1). The median age was 46.00 (38.00–53.00) years, ranging from 21.00 to 77.00 years (Table 1). The proportion of female and liver cirrhosis were 12.62 and 74.42%, respectively. The median sessions of ALSS treatment was 4.0 (3.0–6.0), and 163 (54.2%) patients responded to ALSS treatment. The 90 day transplant-free survival was 62.8%. The median COSSH ACLF score (6.95 vs. 5.99, *p* < 0.001), CLIF-C ACLF score (44.71 vs. 38.05, *p* < 0.001), AARC score (10.50 vs. 9.00, *p* < 0.001), and white blood cell count (8.09 × 10^9^/L vs. 6.45 × 10^9^/L, *p* < 0.001) in mortality patients were significantly higher than that in the others.

**Table 1:** Characteristics of ACLF patients.

	All patients (*N* = 301)	Patients with different stages					
		Pre-stage (*n* = 43)	Early-stage (*n* = 106)	Mid-stage (*n* = 85)	End-stage (*n* = 67)	*χ* ^2^	*p* value
Age (years)	46.00 (38.00–53.00)	44.00 (32.00–48.00)	46.00 (36.00–52.25)	49.00 (39.50–53.00)	46.00 (38.00–55.00)	4.28	0.233
Female	38 (12.62%)	1 (2.33%)^a^	12 (11.32%)^a, b^	11 (12.94%)^a, b^	14 (20.90%)^b^	8.46	0.037
Liver cirrhosis	224 (74.42%)	22 (51.16%)^a^	79 (74.53%)^b^	68 (80.00%)^b^	55 (82.09%)^b^	15.68	0.001
HBV DNA (log10 IU/mL)	4.73 (3.45–6.49)	4.73 (3.19–5.93)	4.56 (3.49–6.57)	4.59 (3.25–6.18)	5.44 (3.57–7.13)	4.56	0.207
Causes of liver disease						1.71	0.635
HBV infection only	227 (75.42%)	33 (76.74%)	78 (73.58%)	68 (80.00%)	48 (71.64%)		
Coexisting with other causes^■^	74 (24.58%)	10 (23.26%)	28 (26.42%)	17 (20.00%)	19 (28.36%)		
Comorbidities^◆^	52 (17.28%)	9 (20.93%)	19 (17.92%)	15 (17.65%)	9 (13.43%)	1.13	0.769
Disease severity assessment							
COSSH ACLF score	6.24 (5.71–6.94)	5.48 (5.24–6.11)^a^	5.92 (5.49–6.38)^b^	6.37 (6.02–6.99)^c^	7.06 (6.64–7.81)^d^	91.76	<0.001
CLIF-C ACLF score	40.17 (35.03–45.18)	34.74 (31.86–39.26)^a^	37.30 (32.13–40.85)^a^	41.86 (38.26–45.91)^b^	45.66 (42.69–49.18)^c^	75.81	<0.001
CLIF-OF score	9.00 (8.00–10.00)	8.00 (8.00–8.00)^a^	8.00 (8.00–8.00)^a^	9.00 (9.00–10.00)^b^	10.00 (10.00–11.00)^c^	200.55	<0.001
AARC score	10.00 (9.00–11.00)	8.00 (8.00–9.00)^a^	9.00 (8.00–10.00)^a^	10.00 (9.00–10.00)^b^	11.00 (11.00–12.00)^c^	99.89	<0.001
Organ function assessment							
Liver (Total bilirubin (μmol/L))	419.20 (332.5–516.45)	388.70 (339.70–460.10)	419.90 (328.33–501.13)	397.40 (321.60–523.25)	443.10 (349.3–529.20)	5.92	0.116
Coagulation (PT-INR)	2.02 (1.66–2.45)	1.41 (1.34–1.45)^a^	1.74 (1.64–1.88)^b^	2.20 (2.11–2.36)^c^	2.84 (2.71–3.17)^d^	285.75	<0.001
Kidney (CLIF-OF score)	1.00 (1.00–1.00)	1.00 (1.00–1.00)	1.00 (1.00–1.00)	1.00 (1.00–1.00)	1.00 (1.00–1.00)	3.58	0.311
Brain (CLIF-OF score)	1.00 (1.00–1.00)	1.00 (1.00–1.00)^a, b^	1.00 (1.00–1.00)^a^	1.00 (1.00–1.00)^b^	1.00 (1.00–1.00)^b^	12.38	0.006
Circulation (CLIF-OF score)	1.00 (1.00–1.00)	1.00 (1.00–1.00)	1.00 (1.00–1.00)	1.00 (1.00–1.00)	1.00 (1.00–1.00)	1.91	0.591
Respiration (CLIF-OF score)	1.00 (1.00–1.00)	1.00 (1.00–1.00)^a^	1.00 (1.00–1.00)^a^	1.00 (1.00–1.00)^a, b^	1.00 (1.00–2.00)^b^	15.75	0.001
White blood cell count (×10^9^/L)	6.78 (5.14–8.96)	6.85 (4.95–8.49)	6.48 (5.10–8.92)	6.83 (5.05–8.92)	7.37 (5.48–10.07)	3.69	0.297
ALSS therapy							
Sessions of ALSS therapy	4.00 (3.00–6.00)	3.00 (2.00–5.00)^a^	4.00 (2.75–6.00)^a, b^	3.00 (2.50–5.00)^a, b^	5.00 (3.00–7.00)^b^	10.01	0.019
Respond to ALSS therapy	163 (54.2%)	29 (67.4%)^a^	53 (64.6%)^a^	65 (55.6%)^a^	16 (27.1%)^b^	24.15	<0.001
90 day transplant-free survival	189 (62.8%)	38 (88.4%)^a^	75 (70.8%)^a, b^	52 (61.2%)^b^	24 (35.8%)^c^	35.88	<0.001

HBV, hepatitis B virus; ACLF, acute-on-chronic liver failure; COSSH, Chinese Group on the Study of Severe Hepatitis B; CLIF-C, European Association for the Study of the Liver—Chronic Liver Failure (CLIF)-Consortium; CLIF-OF, CLIF-organ failure; AARC, APASL ACLF Research Consortium; APASL, Asian Pacific Association for the Study of the Liver; PT-INR, international normalized ratio (INR) of prothrombin time (PT); ALSS, artificial liver support system. Coexisting with other causes^■^: the ones having HBV infection plus any one of other co-existing liver diseases were classified to this subgroup. Comorbidity^◆^: the ones having any one of comorbidities was classified as the comorbidity group. Quantitative data are represented as median (interquartile range) and compared by Mood’s median test. Qualitative data are represented as frequencies (proportion) and compared by chi-squared test. Each superscript letter denotes a subset of patients with different PT-INR whose value do not differ significantly from each other at the 0.05 level.

There were 43 cases of Pre-stage ACLF, 106 of Early-stage ACLF, 85 of Mid-stage ACLF, and 67 of End-stage ACLF (Table 1). No significant difference was seen in age, HBV DNA (log10 IU/mL), and total bilirubin levels in patients with different stages (all *p* > 0.05). The median COSSH ACLF scores of Pre-stage ACLF (5.48), Early-stage ACLF (5.92), Mid-stage ACLF (6.37), and End-stage ACLF (7.06) were significantly different (all adjusted *p* < 0.05). The proportion of patients with End-stage ACLF who responded to ALSS treatment (27.1%) was lower than that of patients with Pre-stage ACLF (67.4%), Early-stage ACLF (64.6%), and Mid-stage ACLF (55.6%) (all adjusted *p* < 0.05). The 90 day transplant-free survival decreased from 88.4% in patients with Pre-stage ACLF to 35.8% in patients with End-stage ACLF (*p* < 0.001).

### Association between stages of ACLF and 90 day mortality

**Figure 2:**
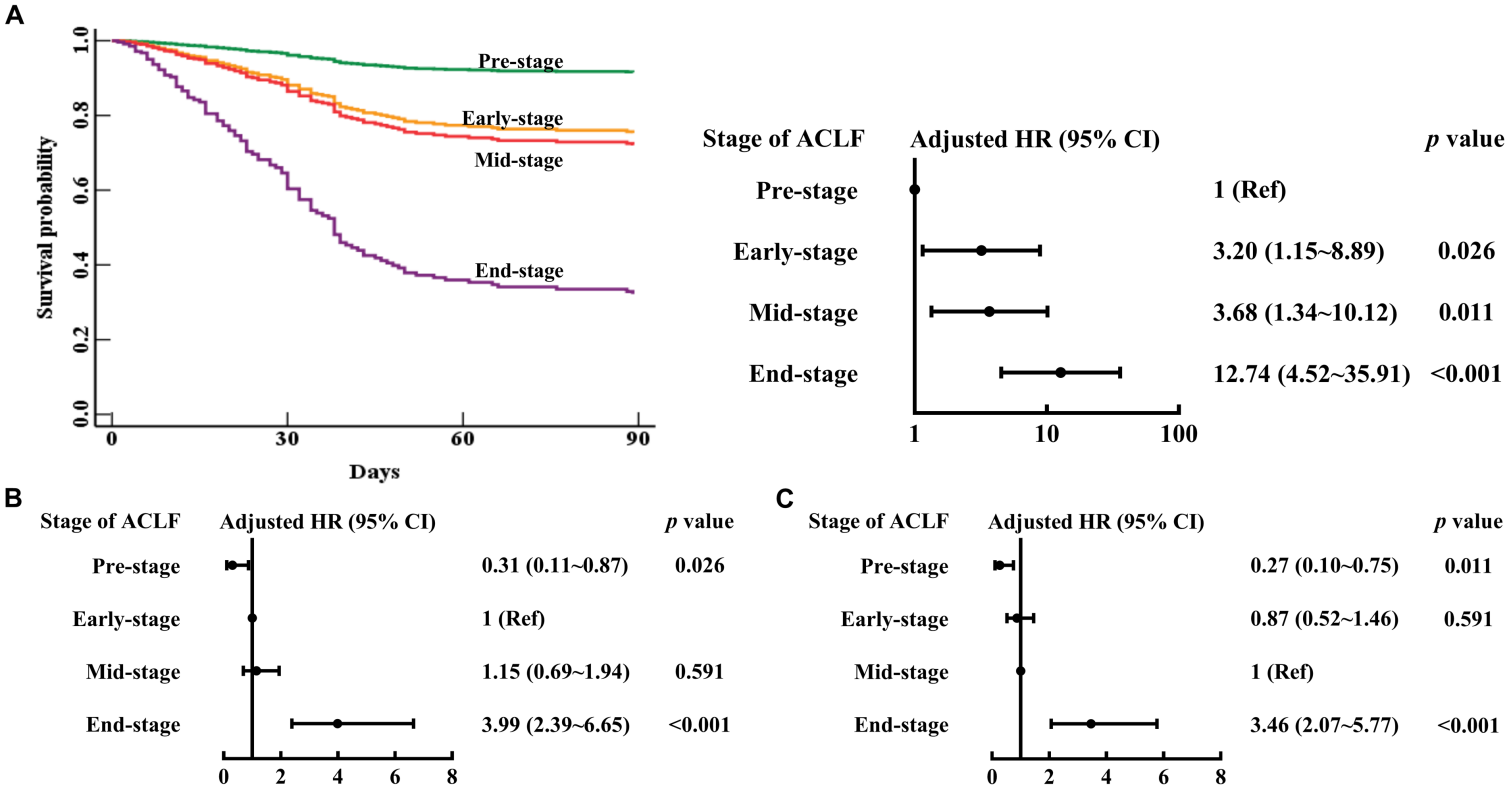
Survival curves and risk of 90 day mortality in patients with Pre-stage, Early-stage, Mid-stage, or End-stage ACLF. ACLF, acute-on-chronic liver failure; HR, hazard ratio; CI, confidence interval. The Pre-stage **(A)**, Early-stage **(B)**, Mid-stage **(C)** are used as the reference. Adjusted HR: multivariable Cox regression analysis includes age (continuous years), gender (female vs. male), liver cirrhosis (yes vs. no), HBV DNA (continuous log10 IU/mL), other co-existing liver diseases, comorbidities, organ function of liver (total bilirubin (μmol/L)), coagulation (stages of ACLF: Pre-stage, Early-stage, Mid-stage, and End-stage), kidney (CLIF-OF score), brain (CLIF-OF score), circulation (CLIF-OF score) and respiration (CLIF-OF score), white blood cell count (×10^9^/L), and sessions of ALSS treatment (continuous data).

As shown in Table 2 and Figure 2, in the multivariable Cox proportional hazards model established with age (continuous years), gender (female vs. male), liver cirrhosis (yes vs. no), HBV DNA (continuous log10 IU/mL), other co-existing liver diseases, comorbidities, organ function of liver (total bilirubin (μmol/L)), coagulation (4 stages of ACLF: Pre-stage, Early-stage, Mid-stage, and End-stage), kidney (CLIF-OF score), brain (CLIF-OF score), circulation (CLIF-OF score) and respiration (CLIF-OF score), white blood cell count (×10^9^/L), and sessions of ALSS treatment, the 90 day mortality risk of Early-stage ACLF patients (adjusted hazard ratio (aHR) (95% confidence interval (CI)), 3.20 (1.15–8.89), *p* = 0.026), Mid-stage ACLF patients (3.68 (1.34–10.12), *p* = 0.011), and End-stage ACLF patients (12.74 (4.52–35.91), *p* < 0.001) were higher than that of Pre-stage ACLF patients. The white blood cell count was an independent risk factor (aHR (95% CI), 1.10 (1.04–1.16), *p* = 0.001).

**Table 2:** Association of stages of ACLF and other factors with 90 day mortality in ACLF patients.

	Multivariate	
	Adjusted HR^▲^ (95% CI)	*p* value
Age (years)	1.03 (1.01–1.05)	0.011
Sex		
Male	1 (Ref)	
Female	1.97 (1.14–3.40)	0.015
Liver cirrhosis		
No	1 (Ref)	
Yes	2.17 (1.18–4.01)	0.013
HBV DNA (log10 IU/mL)	1.14 (1.01–1.28)	0.030
Causes of liver disease		
HBV infection only	1 (Ref)	
Coexisting with other causes^■^	0.90 (0.55–1.47)	0.665
Comorbidities^◆^		
No	1 (Ref)	
Yes	1.96 (1.20–3.19)	0.007
Organ function assessment		
Liver (Total bilirubin (μmol/L))	1.01 (1.01–1.01)	<0.001
Coagulation (Stage of ACLF)		
Pre-stage	1 (Ref)	
Early-stage	3.20 (1.15–8.89)	0.026
Mid-stage	3.68 (1.34–10.12)	0.011
End-stage	12.74 (4.52–35.91)	<0.001
Kidney (CLIF-OF score)	2.29 (1.25–4.20)	0.007
Brain (CLIF-OF score)	3.47 (2.17–5.56)	<0.001
Circulation (CLIF-OF score)	3.05 (1.55–6.03)	0.001
Respiration (CLIF-OF score)	0.72 (0.47–1.13)	0.151
White blood cell count (×10^9^/L)	1.10 (1.04–1.16)	0.001
Sessions of ALSS therapy	0.81 (0.73–0.90)	<0.001

HBV, hepatitis B virus; ACLF, acute-on-chronic liver failure; CLIF-OF, Chronic Liver Failure (CLIF)-organ failure; ALSS, artificial liver support system; HR, hazard ratio; CI, confidence interval. Co-existing with other causes^■^: the ones having HBV infection plus any one of other co-existing liver diseases were classified to this subgroup. Comorbidity^◆^: the ones having any one of comorbidities were classified as the comorbidity group. Adjusted HR^▲^: multivariable Cox regression analysis.

**Figure 3:**
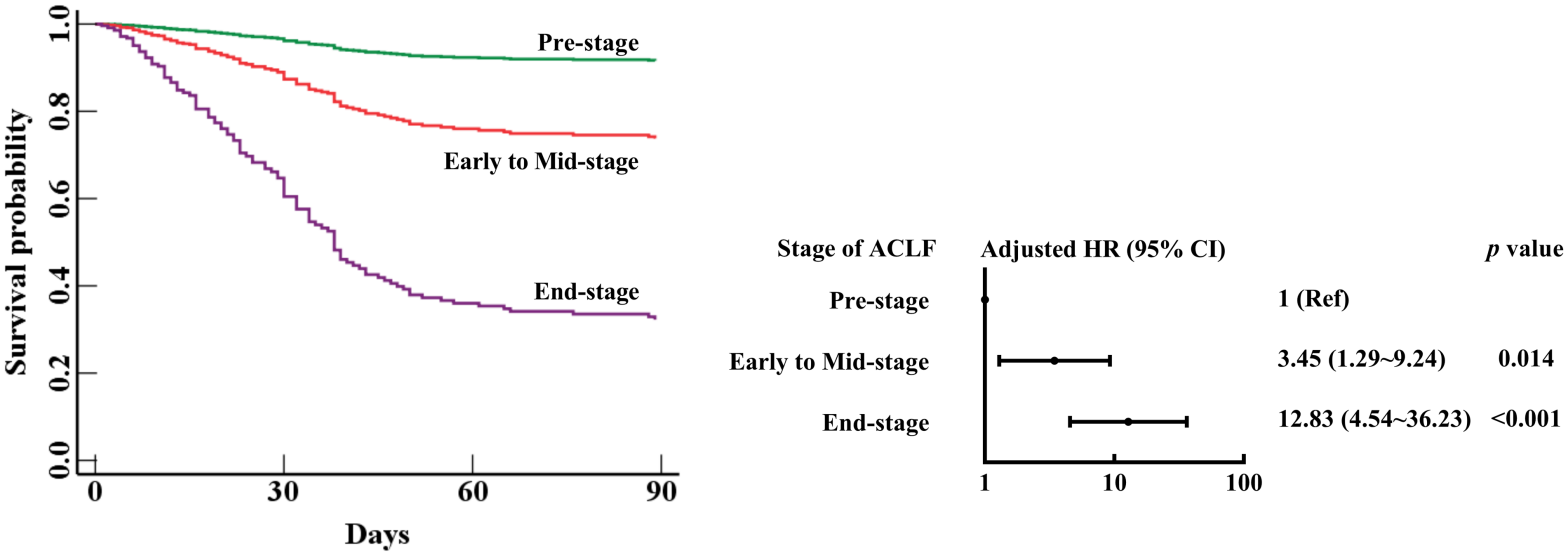
Survival curves and risk of 90 day mortality in patients with Pre-stage, Early to Mid-stage, or End-stage ACLF. ACLF, acute-on-chronic liver failure; HR, hazard ratio; CI, confidence interval. Adjusted HR: multivariable Cox regression analysis includes age (continuous years), gender (female vs. male), liver cirrhosis (yes vs. no), HBV DNA (continuous log10 IU/mL), other co-existing liver diseases, comorbidities, organ function of liver (total bilirubin (μmol/L)), coagulation (stages of ACLF: Pre-stage, Early to Mid-stage, and End-stage), kidney (CLIF-OF score), brain (CLIF-OF score), circulation (CLIF-OF score) and respiration (CLIF-OF score), white blood cell count (×10^9^/L), and sessions of ALSS treatment (continuous data).

The 90 day mortality risk of Mid-stage ACLF patients was similar to that of Early-stage ACLF patients (aHR (95% CI), 1.15 (0.69–1.94), *p* = 0.591) (Figure 2). The 90 day mortality risk of Early to Mid-stage ACLF patients was higher than that of Pre-stage ACLF patients (aHR (95% CI), 3.45 (1.29–9.24), *p* = 0.014) (Figure 3).

### Association between sessions of ALSS treatment and 90 day mortality

As shown in Table 2, in the multivariable Cox proportional hazards model established above, the sessions of ALSS treatment was an independent protective factor (aHR (95% CI), 0.81 (0.73–0.90), *p* < 0.001).

**Figure 4:**
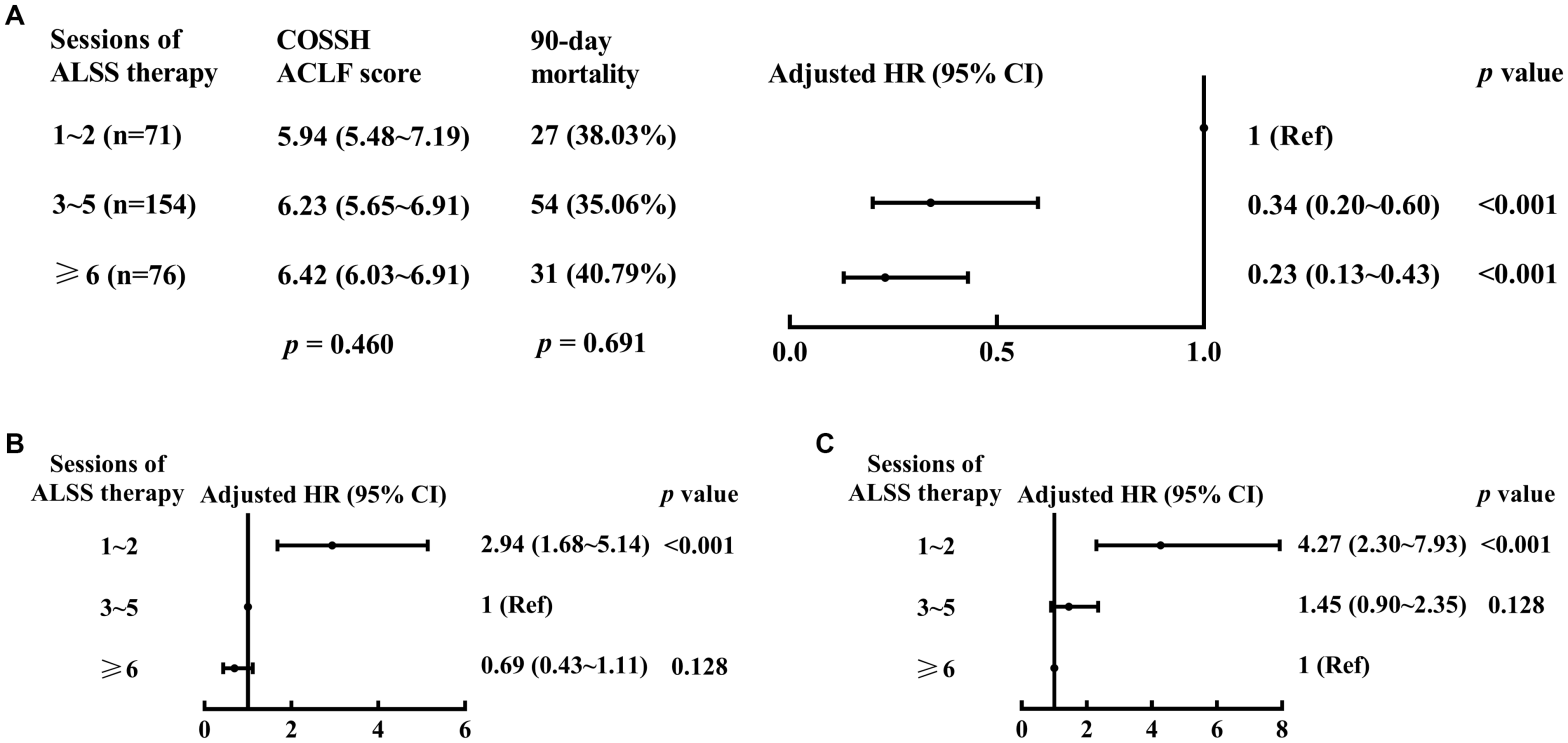
90 day mortality risk of different sessions of ALSS treatment for ACLF patients. ALSS, artificial liver support system; ACLF, acute-on-chronic liver failure; HR, hazard ratio; CI, confidence interval. The 1–2 **(A)**, 3–5 **(B)**, or ≥6 **(C)** sessions of ALSS treatment are used as the reference. Adjusted HR: multivariable Cox regression analysis includes age (continuous years), gender (female vs. male), liver cirrhosis (yes vs. no), HBV DNA (continuous log10 IU/mL), other co-existing liver diseases, comorbidities, organ function of liver (total bilirubin (μmol/L)), coagulation (4 stages of ACLF: Pre-stage, Early-stage, Mid-stage, and End-stage), kidney (CLIF-OF score), brain (CLIF-OF score), circulation (CLIF-OF score) and respiration (CLIF-OF score), white blood cell count (×10^9^/L), and sessions of ALSS treatment (≥6 sessions vs. 3–5 sessions vs. 1–2 sessions).

In a similar multivariable Cox proportional hazards model established with stages of ACLF (4 stages: Pre-stage, Early-stage, Mid-stage, and End-stage), sessions of ALSS treatment (≥6 sessions vs. 3–5 sessions vs. 1–2 sessions), and the others mentioned above, the 90 day mortality risk in patients treated with 3–5 sessions of ALSS treatment (aHR (95% CI), 0.34 (0.20–0.60), *p* < 0.001) or ≥6 sessions (0.23 (0.13–0.43), *p* < 0.001) were lower than that of patients treated with 1–2 sessions, respectively (Figure 4). Although the 90 day mortality risk in patients treated with ≥6 sessions of ALSS treatment was similar to that of patients treated with 3–5 sessions (aHR (95% CI), 0.69 (0.43–1.11), *p* = 0.128), End-stage ACLF patients treated with ≥6 sessions of ALSS treatment had lower 90 day mortality risk than those treated with 3–5 sessions (aHR (95% CI), 0.40 (0.18–0.92), *p* = 0.031).

## Discussion

In this retrospective cohort study, we found staging of PT-INR was an independent risk factor for 90 day mortality in ACLF patients received ALSS treatment. Compared to those in End-stage, the Pre-, Early- and Mid-stages ACLF patients who had lower 90 day mortality risk were more eligible for ALSS treatment. ACLF patients received 3–5 sessions of ALSS treatment had lower 90 day mortality risk.

Liver is a vital organ which performs important physiological functions such as metabolism of carbohydrates, lipids, and proteins, detoxification of bilirubin, ammonia and xenobiotics, and synthesis of bile acids and blood coagulation factors (26, 27). When the liver function is damaged, the decrease in the amount as well as the function of coagulation factors will result in a variety of coagulation dysfunction, and the PT-INR will be prolonged correspondingly (28, 29). Previous studies have reported that PT-INR is an independent risk factor closely related to disease severity and prognosis of ACLF patients (1–3). In this study, we found staging of PT-INR was an independent risk factor for 90 day mortality in ACLF patients. The 90 day mortality risk of Pre-, Early-, and Mid-stages ACLF patients (1.3 ≤ PT-INR < 2.5) were lower than that of End-stage ACLF patients (PT-INR ≥ 2.5). Similarly, Zeng YY and his colleagues reported ACLF patients with PT-INR <2.6 had lower 90 day mortality than those with PT-INR ≥ 2.6 (33.44% vs. 80.60%, *p* < 0.001) (30). In their study, they took 2.5 for the best cutoff value of PT-INR in predicting 90 day mortality of ACLF patients received ALSS treatment (30). It endows the stages of ACLF with some rationality to use in our study. In fact, our staging of PT-INR is consistent with the cutoff values of coagulation in CLIF-OF score (1). Recently, two predictive models, namely sMELD score and PALS score, have been established using similar cutoff values of PT-INR and are considered to have good predicative values in predicting 90 day mortality of patients with HBV-ACLF received ALSS treatment (17, 31).

Accurate prediction of the prognosis of ACLF patients is helpful for clinical decision-making and treatment selection. Previous studies have shown that ACLF patients with higher COSSH ACLF score, CLIF-C ACLF score, CLIF-C OF score, AARC score, or Model for End-Stage Liver Disease (MELD) score had poorer prognosis (1–3). The COSSH ACLF score was considered to be a more accurate model in predicting short-term prognosis in HBV-ACLF patients (3), and those with COSSH ACLF score ≤ 6.59 had good response to ALSS treatment (32). However, the disadvantage of tedious calculation in COSSH ACLF score makes it un-convenient for clinical application. The stages of ACLF used in this study was simplified. The Pre-, Early-, and Mid-stages ACLF patients responded to ALSS treatment well. In addition, we found the 90 day mortality risk of Mid-stage ACLF patients (2.0 ≤ PT-INR < 2.5) was similar to that of Early-stage ACLF patients (1.5 ≤ PT-INR < 2.0). This finding is consistent with the previous result that HBV-ACLF patients with 1.5 ≤ PT-INR < 2.5 belong to the ACLF-1 group and has similar prognosis (3). Taken together, the stages of ACLF could be used at bedside as a preliminary method for clinical screening, and ACLF patients in Pre-, Early- and Mid-stages (PT-INR < 2.5) might be more eligible for ALSS treatment.

ALSS treatment could work as a bridge to recovery or liver transplantation for ACLF patients (12, 33). The intensity of ALSS treatment is one of the central issues of clinical practice (34). Univariate analysis of a previous study showed that HBV-ACLF patients received ≥3 sessions of PE-centered ALSS treatment had lower 90 day mortality (17). Similarly, a meta-analysis of pooled individual-patient data of albumin dialysis in ACLF patients showed that higher treatment intensity (≥5 sessions) was associated with significantly higher survival rates (35). In our study, multivariate analysis found the sessions of ALSS treatment was an independent protective factor for ACLF patients: 3–5 sessions of ALSS treatment could significantly reduce the 90 day mortality risk, while ≥6 sessions might help to improve prognosis in severe cases. A prospective longitudinal cohort study reported that the 90 day period following the occurrence of ACLF was a critical time point for long-term prognosis, with favorable outcomes observed beyond this time frame (36). These results could support the application of 3–5 sessions of ALSS treatment as an early and proactive treatment in most ACLF patients. ACLF patients who do not respond to ≥6 sessions of ALSS treatment should be scheduled for liver transplantation in time. It was reported that ACLF patients received liver transplantation within 30 days of placement on the waitlist would reach over 90% of graft survival probability at 5 years (37).

This study has several limitations. First, all the patients in our study had chronic HBV infection, the results might not be applicable to ACLF patients without HBV infection. Second, as a secondary data analysis based on a previous retrospective, single-center and non-large sample study, the selection bias is inevitable. The patient characteristics might not be representative of the general population, and some potential confounders might be missed and then would cause the significance to be overestimated. Third, we did not include patients with similar conditions who did not receive ALSS treatment for comparative analysis, which might affect the results. Fourth, we only collected baseline data and follow-up patients, and did not collect data pre- and post-ALSS treatment, which failed to fully show the changes in patient’s condition. However, many studies have confirmed the significant changes in liver function pre- and post-ALSS treatment, especially the change in total bilirubin level (38, 39). Finally, although the staging of ACLF used in this study has some rationality (1, 3, 18), it requires more validation.

In conclusion, we found ACLF patients in Pre-, Early-, and Mid-stages might be more eligible for ALSS treatment. Application of 3–5 sessions of ALSS treatment for ACLF patients might be reasonable. In the future, prospective cohort studies should be conducted to validated our findings, and multicenter randomized controlled trials should be conducted to evaluate whether ACLF patients in Pre-, Early- and Mid-stages could definitely benefit from ALSS treatment in order to avoid overtreatment. The mechanism of action of ALSS treatment and its impact on different patient populations also remain to be clarified.

## Data availability statement

The raw data supporting the conclusions of this article will be made available by the authors, without undue reservation.

## Ethics statement

The studies involving humans were approved by the Biomedical Research Ethics Committee of West China Hospital of Sichuan University. The studies were conducted in accordance with the local legislation and institutional requirements. Written informed consent for participation in this study was provided by the participants’ legal guardians/next of kin.

## Author contributions

YM: Conceptualization, Data curation, Formal analysis, Writing – original draft, Writing – review & editing. YX: Data curation, Formal analysis, Writing – original draft. LD: Data curation, Formal analysis, Funding acquisition, Project administration, Resources, Writing – original draft, Writing – review & editing. LB: Conceptualization, Funding acquisition, Resources, Supervision, Writing – review & editing. HT: Conceptualization, Funding acquisition, Resources, Supervision, Writing – review & editing.
